# A Review of the Natural History and Laboratory Culture Methods for the Yellow Dung Fly, *Scathophaga stercoraria*

**DOI:** 10.1673/031.010.1101

**Published:** 2010-03-02

**Authors:** WU Blanckenhorn, AJ Pemberton, LF Bussière, J Roembke, KD Floate

**Affiliations:** ^1^ Zoological Museum, University of Zürich-Irchel, 34-J-98, Winterthurerstrasse 190, CH-8057, Switzerland; ^2^ School of Biological and Environmental Sciences, University of Stirling, Stirling, United Kingdom FK9 4LA; ^3^ ECT Oekotoxikologie GmbH, Böttgerstr. 2–14, D-65439 Flörsheim, Germany; ^4^ Lethbridge Research Centre, Agriculture and Agri-Food Canada, 5403 1st Avenue S, Lethbridge, Alberta, Canada TIJ 4B1

**Keywords:** bioassay, eco-toxicology, faecal residues, life history, non-target effects

## Abstract

The yellow dung fly *Scathophaga stercoraria* (L.) (Diptera: Scathophagidae) is a widespread and locally abundant fly associated with the dung of large mammals, especially farm animals. This species has recently become a standard test organism for evaluating toxic effects of veterinary pharmaceuticals in livestock dung. In this context, a review of its natural history and a general description of the field and laboratory rearing methods of this species are provided here to benefit the scientific community as well as government regulators and applicants of eco-toxicological studies. For guidance, means and ranges are included for all relevant standard life history traits stemming from previously published data on Swiss populations.

## Introduction

The yellow dung fly *Scathophaga stercoraria* (L.) (Diptera: Scathophagidae) is a widespread and locally abundant fly associated with the dung of large mammals. The species was first named by Linnaeus as *Musca stercoraria* in 1758. In 1800, Meigen renamed the genus as *Scopeuma*, and in 1803 he reclassified the species, together with several relatives, into a new family *Scathophagidae*. For unknown reasons, from 1805 onwards, Fabricius used the genus spelling *Scatophaga* for several of the related species (but not *S. stercoraria*, which remained *Scopeuma* for a while). There has been confusion about the spelling of the whole family group, but the spelling was officially settled to become *Scathophaga* in all the standard catalogues (see Gorodkov 1986).

In the early part of the last century, the predatory *S. stercoraria* first attracted interest as a possible bio-control agent of pest flies affecting livestock (Cotterell 1920). During the past 40 years, this fly has been the subject of numerous studies on mating behavior and sperm competition (Parker 1970a,b,c, 1978), post-copulatory sexual selection and sexual conflict (Ward 2000; Hosken et al. 2001), reproductive physiology (Hosken and Ward 1999; Reim et al. 2006), foraging (Blanckenhorn and Viele 1999), life history evolution (Blanckenhorn 1998a,b; Teuschl et al. 2007), thermal biology (Blanckenhorn and Llaurens 2005), developmental stability and fluctuating asymmetry (Strong and James 1992; Swaddle 1997; Hosken et al. 2000; Webb et al. 2007, Floate and Coghlin 2010), phylogenetics (Bernasconi et al. 2001), quantitative genetics (Blanckenhorn 2002), and population genetics (Kraushaar et al. 2002). *S. stercoraria* also have been used to test for non-target effects of chemical residues in dung of livestock treated with veterinary pharmaceuticals (e.g. Sommer et al. 1992; Strong and James 1992; Floate 1998, 2007; Webb et al. 2007, Floate and Coghlin 2010). In this latter context, the international community has approved *S. stercoraria* as a standard test species to evaluate the toxicity of drug residues in livestock dung (OECD 2008). As a result, tests using *S. stercoraria* will become a requirement for the registration of new veterinary compounds (e.g., Römbke et al. 2009).

No papers specifically written to provide information on the rearing of *S. stercoraria* in laboratory culture exist in the literature. Such information may facilitate future studies on the insect's biology and ecology, but is particularly needed for studies in response to the new regulatory requirements for novel livestock medical products. Here a general description of the biology, life history, ecology, and behavior of *S. stercoraria* is provided, including control and reference test data relevant for the assessment of toxicological tests. Subsequently, laboratory rearing and handling methods plus some associated field methods are described. In this review, some original information is included on pupal survival and standard life history traits relevant for the conductance of ecotoxicological applications, which due to availability primarily reflect the situation of Swiss populations. Blanckenhorn (2009) more completely covers the *S. stercoraria* literature.

### General biology, distribution, phenology, behavior and life history

**Figure 1:**
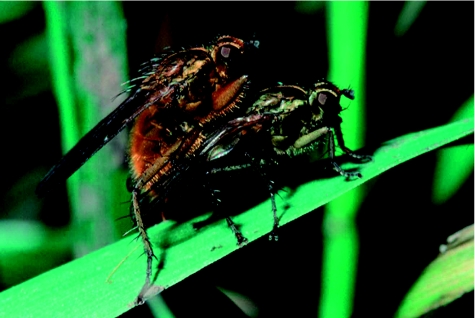
Copulating pair of *S. Stercoraria*. Photo by Peter Jann. High quality figures are available online.

Adult *S. stercoraria* flies are between 7 and 13 mm long. The males are hairy and yellow to orange in color, whereas the females are much less hairy, greenish and more cryptic. Atypically for insects, the males are considerably larger than females (Kraushaar and Blanckenhorn 2002), and unlike most Scathophagids, the sexes differ quite starkly in color (Figure 1). The species inhabits temperate regions of the entire northern hemisphere (Stone et al. 1965; Gorodkov 1986), favoring cooler climates at high altitudes such as the Swiss Alps (Blanckenhorn 1997) and high latitudes up to Iceland or even Spitzbergen (Sigurjónsdóttir and Snorrason 1995). Its distribution appears limited by hot temperatures toward the south (Hammer 1941; Ward and Simmons 1990; Blanckenhorn 1998a, Blanckenhorn et al. 2001), where the flies occur only at higher elevations, such as the Pyrenees and Sierra Nevada in Spain or the Sierras of Mexico (Stone et al. 1965). *S. stercoraria* is also reported from South Africa, although this is probably a closely related sister species, *S. soror* (Werner et al. 2006). It is not reported from anywhere else in the southern hemisphere. In North-Central Europe, *S. stercoraria* is one of the most abundant and widespread insect species associated with cow dung, although some similar scathophagid species may co-occur on cattle pastures, depending on location, but usually at much lower densities (e.g. *S. furcata* in North America and *S. inquinata, S. suilla*, and *S. lutaria* in Europe). The species' distribution pattern is likely influenced by human agricultural practices. While *S. stercoraria* is considered a cow-dung specialist, it can successfully breed on dung of other large mammals such as sheep (Hirschberger and Degro 1996), horse, deer, or wild boar (Blanckenhorn et al., unpublished data).

*S. stercoraria* larvae are coprophagous, feeding on the dung of large mammals, which they thereby help to decompose along with many other species of competing earthworms, beetles and flies (Hammer 1941; Holter 1979). Adult *S. stercoraria*, in contrast, are sit-and-wait predators of small insects, but they also imbibe nectar and fresh dung as energy sources (Cotterell 1920; Hammer 1941; Gibbons 1980a,b; Sasaki 1984). Adult flies are nutritionally anautogenous (or capital breeders) requiring protein and lipids from prey to become sexually mature producers of eggs and sperm (Foster 1967).

*S. stercoraria* phenology depends strongly on local climate. In Central Europe, fly populations often exhibit a sharp population decline in summer (Hammer 1941; Parker 1970; Gibbons 1987; Blanckenhorn 1997; Jann et al. 2000; Blanckenhorn et al. 2001), subdividing the year into a spring (March — June) and an autumn (September — November) flight season. The summer decline is mediated by temperatures above 25° C, which tend to kill larvae, pupae and adults. Adult flies avoid this by moving into cooler, forested microhabitats and entering some sort of physiological quiescence (Gibbons 1987; Ward and Simmons 1990; Blanckenhorn 1998a; Blanckenhorn et al. 2001). In contrast, the flies are present only in summer in the highlands of the Swiss Alps (1500–2800 m) (Blanckenhorn 1997) and in northern European countries such as Iceland and Finland (Sigurjónsdóttir and Snorrason 1995; Otronen 1996). Accordingly, the number of generations per year varies with latitude and altitude, with two to three (overlapping) generations per year in lowland Switzerland (Jann et al. 2000), and one or two generations per year in the Swiss highlands and Northern Europe. The flies diapause over winter in the pupal stage.

*S. stercoraria* females spend most of their time foraging for prey and nectar in the vegetation surrounding the pasture and only come to the dung to oviposit. Males also need to forage in the surroundings but spend most of their time waiting on or around fresh dung pats to mate with incoming females (Parker 1970b). During copulation, which typically lasts about 20–50 minutes (though there is considerable variation), and during the ensuing oviposition, the male guards the female against competitors so his sperm are not displaced before oviposition (Parker 1978; Simmons 2001). Competition among males for females is typically intense, as the operational sex ratio at the dung is highly male biased; a single dung pat may host as many as 400 males (Jann et al. 2000). After copulation, the female lays about 30–90 eggs into the dung and then leaves the pat for further foraging in the vegetation. The male waits for other females at the same pat or switches to another, fresher pat, as pats lose their attractiveness to this fresh dung specialist within 1–2 h of being deposited (Parker 1970a).

Larvae hatch from the eggs within 1–2 days, depending on temperature, and immediately enter the dung to avoid desiccation or drowning. Thereafter, they regularly surface for oxygen. Other sources of egg, larval and pupal mortality in the dung community include numerous egg and larval predators such as staphylinid beetles and hymenopteran parasitoids (Hammer 1941; Sowig et al. 1997). Larvae undergo three molts and, at 20° C, grow exponentially and rapidly during the first five days of development; thereafter they require an additional five days to empty their guts and prepare for pupation, during which time no additional body mass is accumulated (Blanckenhorn 1999; Teuschl et al. 2007).

Individuals pupate in the encrusted parts of the dung or in the ground under or near the dung pat. Pupal development takes an additional 10 days at 20° C. In the case of direct (i.e. non-diapause) development, adult flies emerge after a total pre-adult (egg + larval + pupal) development time between 17 days (at 25° C) and 80 days (10° C and below; Table 1). On average, the smaller females emerge a few days earlier.

### Collecting wild *S. stercoraria* in the field

*S. stercoraria* can be easily collected while mating on a pasture at the appropriate time in the season (see above). Overcast conditions (no rain), mild weather (not too hot) and certain times of day (the last two hours before sunset) usually work best. A sweep-net can be used, although copulating pairs (as well as single males) are more efficiently caught by carefully lowering a long (e.g. 50 ml) glass or plastic vial over the insects while they copulate on or near the dung and then plugging it with a permeable paper or foam stopper. Live single flies or pairs then can be brought back to the lab in these vials, to which a bit of sugar and/or moist paper or cotton is best added, preferably in a dark box under mild refrigeration. Alternatively, freshly laid eggs can be scooped with a small spatula directly into a plastic vessel containing fresh dung and plugged with a stopper, wherein the larvae can subsequently hatch and develop in the laboratory. Emerging adults then can be collected as described in the egg processing section below. If the flies are not needed alive for procedures such as molecular analyses, individuals can be transferred directly into pure ethanol or can be frozen in the lab and subsequently stored in ethanol.

### Laboratory holding conditions

Adult *S. stercoraria* are cannibalistic in the absence of alternate prey, as they require prey to produce eggs and sperm (Foster 1967). Cannibalism can be prevented by keeping flies individually in glass or plastic culture bottles (100 ml volume, for example) capped with paper or foam stoppers. Stoppers should fit the bottles snugly to prevent phoretic and parasitic mites from moving between bottles. Each bottle should be equipped with a water source (e.g. gauze or cotton submerged in water in a small dish), plus sugar in a separate dish; diluted sugar water is also suitable. Rearing conditions not higher than 24° C with humidity of 50–60% and a light period of 12–14 h are recommended.

Ideal prey include flies smaller than dung flies. Mass-reared *Drosophila* spp. or *Musca domestica* work well, both of which are commercially available and cultured in many laboratories. Start freshly emerged *S. stercoraria* with *ad libitum* amounts of ca. 50 *Drosophila melanogaster* per week (or equivalents thereof) and re-feed when all prey are eaten. Otherwise, feed every week or even two weeks, making sure that the water does not dry up. After attaining sexual maturity (Table 1), males will often stop eating prey while females will require new prey to replace each clutch of eggs laid. Ten *Drosophila melanogaster* per week will limit reproductive output (Jann and Ward 1999). Holding bottles tend to become filthy and moldy after about one month, when they should be replaced. All bottles, stoppers and containers for food and water need to be disposed of, or carefully cleaned, to avoid mite infestations within the lab culture. For material that is not easily washed (such as paper stoppers), autoclaving in an oven at 120° C is adequate to prevent infestations.

Holding flies individually in bottles is time-consuming and requires considerable laboratory space. When doing so, subsequent generations can be produced by mating a number (for example, *n* = 30) of randomly chosen pairs and raising their offspring, typically from the first-laid (partial) clutch, in disposable containers as described below and keeping a subset of the emerging adults to be further propagated. As an alternative, *S. stercoraria* can be held in groups in large, well-aerated cages. *S. stercoraria* colonies have been maintained for several years in plexiglass cages (60 cm × 60 cm × 60 cm), each housing several hundred flies allowed to feed *ad libitum* on suitable prey with access to water and sugar. Lower densities of about five flies per litre (= 10 cm × 10 cm × 10 cm) can be used to optimize production, with the sexes initially separated. Otherwise, males will harass non-receptive females and ultimately delay reproduction.

**Table 1:**
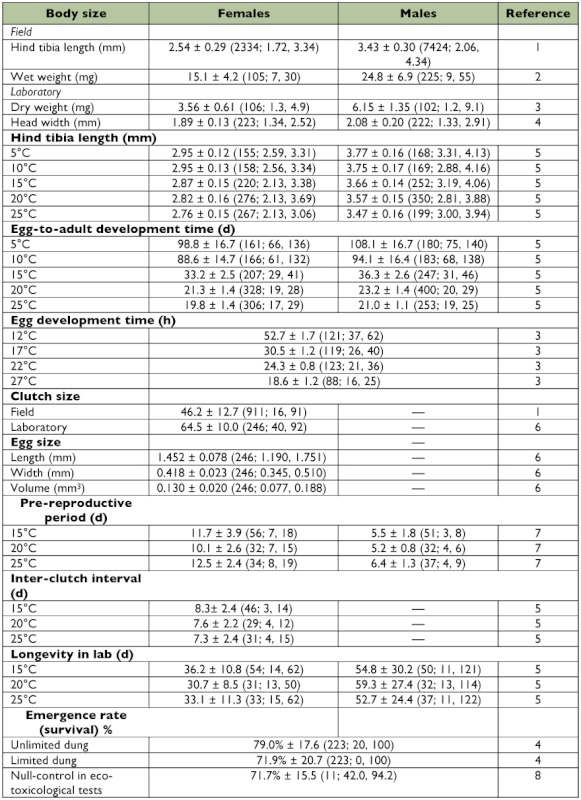
Mean ± SD (n; min, max) life history traits for Swiss S. stercoraria at various conditions (laboratory data unless specified).

**continued:**
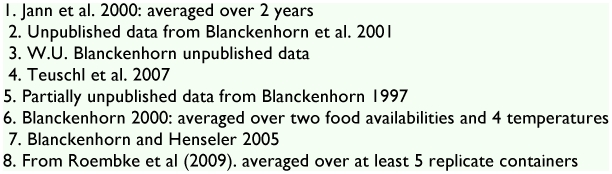

For ease of colony maintenance when flies are housed in large cages, dung pats (ca. 1 litre) on Styrofoam plates with an underlying layer (ca. 1 cm thick) of sand and vermiculite (1:1) can be placed into the cage with the adult flies. This can be started approximately 2–3 weeks after the first emergence of adults to allow time for sexual maturation and mating. After 2–3 days of exposure to ovipositing females, the dung pat is removed and replaced with a fresh one, if more eggs are needed. The Styrofoam plates with the dung containing eggs and larvae are subsequently held for 2–3 weeks in a plastic tub for larval development and pupation. The plate is elevated above the floor of the tub by placing it on a Petri dish. The tub is then almost fully covered with a sheet of plastic to reduce dehydration of the dung. However, one corner of the tub should be left uncovered. Otherwise, the heat generated by larval metabolism will cause the tub to overheat and reduce larval survival. Larvae will typically reduce the dung pat to a granular consistency, which is readily sieved to remove pupae. Larvae also will crawl off the plate to pupate underneath. If the plate is not elevated, there is insufficient space for pupation, in which case pupae are often deformed and adult emergence is low. Each dung pat will produce from a few dozen to several hundred adult flies. Pupae removed from tubs can be placed in shallow Petri dishes in clean cages for emergence of the adults.

In general, when handling *S. stercoraria* adults, keep in mind that they are positively photo-tactic, and, therefore, they are best moved from one into another container, or kept in a particular bottle, by orienting the opening toward or away from the light, respectively. Thus it is best to work at a window or facing a strong lamp (at night) with overhead lighting switched off.

### Adult maturation and reproductive period

Newly emerged *S. stercoraria* females take about 2 weeks (10–16 d) and males about 1 week (5–8 d) to become sexually mature when held individually and given an excess of sugar, water and prey (Table 1; Gibbons 1980a; Blanckenhorn and Henseler 2005). Although females will mate after about 6 d, they usually require at least 10 d to produce a batch of eggs. The abdomens of fully gravid females look swollen and whitish when viewed from the side or below. Inter-clutch intervals are between 3–7 days in the laboratory, depending on nutrition and temperature (but probably are considerably longer in the field; cf. Gibbons 1987). After first producing sperm, males can and will mate at least 5 times, indeed more or less continuously if their sperm stores are refilled by having continuous access to prey. Under (benign) laboratory conditions, flies live, on average, for 1–2 months, up to a maximum of several months, during which females can produce 10 clutches or more. Females store sperm from each copulation, which should last to fertilize at least 4 clutches, so repeated mating is not strictly necessary, although a recent mating will increase the likelihood of oviposition when females are offered dung. Also, gravid females may dump fertilized or unfertilized eggs into the holding bottle if not given an opportunity to lay.

### Laboratory matings

When males and females are sexually mature, matings of individually-held flies are best staged in a 50 or 100 ml vial containing a smear of dung on wet filter or blotting paper. Males will also mate without dung, but the presence of dung is believed to stimulate copulation, although this has not been tested rigorously. To reflect the prior arrival of males at the dung in nature, the male is typically placed in the vial first, and then female is added. Copulations can begin almost instantly, or there can be a delay of a few minutes up to one hour. Some pairs may never mate, in which case the male should be exchanged after 10 to 15 min. For experimental matings, which last between 20 and 50 min, the following information can be easily recorded: (1) the time the female was added, (2) the time copulation started, and (3) the time copulation ended. These give measures of ‘latency’ to copulate (2 minus 1) and copulation duration (3 minus 2). After copulation ends (i.e. the male and female abdomens have become detached), males may guard (i.e. stand over the female without genital contact) while the female deposits her eggs into the dung, which takes about 20 min. However, some males may not guard the females in the vials used for laboratory copulations, and they will dismount and then likely copulate again with the same female (presumably not recognizing her identity); thus males should be removed after copulation to avoid further harassment. When re-mating non-virgin, gravid females, there is a risk that she will start ovipositing into the dung before the new male copulates. This risk can be reduced by only providing dung after the focal copulation terminates.

**Figure 2:**
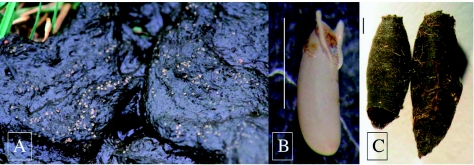
(A) *S. stercoraria* eggs in a dung pat; (B) close-up of one egg, with the yellowish plastron and the respiratory horns clearly visible; and (C) two pupae (the left one is open after the emergence of an adult). Scale bars are 1 mm. Photos by Peter Jann, Stephanie Bauerfeind and Marlen Wildenhues. High quality figures are available online.

### Egg processing

*S. stercoraria* eggs are white and banana-shaped, similar to, but larger than, the eggs of *Drosophila melanogaster* (Figure 2A, B; Table 1). Females will stick the eggs lengthwise (vertically) into the dung singly or in groups, so that only the upper pole will be exposed to the air, the rest being submerged. The upper pole features a yellowish flap (plastron), through which the larva will eventually exit, flanked by two little lateral respiratory horns (Figure 2B; Arthur et al. 2008).

Individual females will typically lay entire clutches of eggs (30–90 eggs, depending on body size; Table 1; Blanckenhorn 2009), although they sometimes only deposit a partial clutch. After oviposition into the dung portion on filter paper, the female can be transferred to another vial and the filter paper with the eggs removed to be counted *in situ* under a binocular microscope. Using curved, smooth forceps, as many eggs as necessary can then be transferred face up (in groups, with a bit of dung) into a disposable plastic container with excess fresh dung (i.e. > 2 g per individual, Amano 1983) for propagation of the next generation, to be capped with a paper or foam stopper or paper towel fastened with rubber bands. This should be done quickly (within several hours) after egg deposition to avoid excessive drying of the dung surface, which can make the extraction of intact eggs difficult. The respiratory horns should not be submerged below the dung surface. Also, the containers should be sufficiently large (e.g. 100 ml) and only about half full of dung, leaving some space for emerged adults.

To measure the size of eggs, and to easily score larval hatching success (e.g. when conducting eco-toxicological tests), single eggs can be randomly removed from the clutch using a small, wetted paint brush, to be placed horizontally on a piece of wet filter paper, brushing off most of the excess dung. Length and width (as egg height and width are roughly the same) of 3–5 randomly chosen eggs of a given clutch are typically measured using an ocular eyepiece or by taking digital photos and then measuring with image analysis software (e.g. ImageJ, http://rsb.info.nih.gov/ij). The volume of eggs can be estimated using the formula for an ellipsoid:

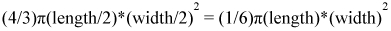


After measuring, the wet filter paper with the eggs is placed onto the (wet) dung in a disposable plastic container as described above. After about 24 h (at room temperature) the larvae will have hatched and crawled into the dung, and deflated egg cases are easily distinguished from the remaining intact (infertile) eggs. Egg development time, i.e. the time from egg deposition to larval hatching, can thus be scored by checking for hatched larvae at regular intervals (e.g. hourly).

When eggs are transferred into a refrigerator at 2–5° C immediately after laying, larval hatching can be delayed for 2–3 d. This is necessary, for example, when eggs are to be sent to a laboratory for immediate ecotoxicological testing. Thus, individual clutches in the dung portion on a round filter paper can be placed in Petri dishes of corresponding size, stacked, and sent via overnight express mail in a styrofoam box containing padding and an ice pack for cooling.

### Collecting emerged flies and scoring development time, emergence success, and body size

As for any ectothermic organism, egg-to-adult development time (as well as body size) is highly temperature dependent (Blanckenhorn 1997; Table 1). It can take as little as 17 d (above 23° C), or about 21 d at 20° C, 35 d at 15° C, and ca. 75 d at 10° C, although at the latter, cooler temperatures some flies will go into winter diapause as pupae (Blanckenhorn 1998b). About half of the development period is spent as a larva, the other half as a pupa (Blanckenhorn 1999; Teuschl et al. 2007). Emergence occurs over several days for a whole clutch (family) reared together in the same dung container, the smaller females emerging first. Egg-to-adult development time is obtained by checking containers for emerging adults on a daily basis. Emergence success (= egg-to-adult survival) is simply the proportion of adults emerged relative to the number of eggs entered or larvae hatched (to be written on the container). Mean emergence success is 60–80% at optimal conditions in the lab (Table 1). Somewhat higher survival can be obtained when raising eggs individually in small dung containers, thus avoiding larval competition, but this is laborious.

When checking emergence, adults can be collected daily from the larval family dung containers and immediately frozen for later measurement of body size, or alternatively transferred into a holding bottle for further propagation, as described above. In principle, any morphological structure can be used as a measure of body size, as most body structures covary positively with one another. However structures composed of individual exoskeletal elements (e.g., a segment of a leg) are most consistent because they do not change size with body condition or female gravid state. Hind tibia length (cut off the leg at the femur) is traditionally used in this species (Parker 1978; Simmons 2001), but teneral fresh (wet) weight or dry weight (after 24 h of drying at 60° C) of freshly emerged adults is also good (Table 1). When measuring live animals, wet weight or head width (= widest extension of head including eyes) are best and should be obtained from individuals anaesthesized with CO2 while measuring.

Alternatively, when simply interested in collecting emerged adults without scoring, the small (e.g. 100 ml) plastic dung container with the developing offspring can be placed open into a larger (e.g. 1.5 liter plastic) container, to be capped with a paper towel lid held with rubber bands. If some sugar and water is added, the flies will survive for a couple of days as a group and hopefully will not eat each other before being processed further.

### Collecting larvae or pupae for further processing

Newly hatched larvae can be collected with a wet paint brush if eggs are processed via the filter-paper technique described above, provided they received no dung; otherwise, it is cumbersome, but possible, to dig out larvae from the dung. Alternatively, single eggs (e.g. for molecular analysis of single larvae) can be transferred directly into wells of a 96-well plate, sealed with sealing foil, and the whole plate can be frozen at -80° C when larvae have hatched.

Because *S. stercoraria* spend about 10 d in the hardy pupal stage, pupae are easy and inexpensive to send using regular mail, in contrast to eggs (as explained above) or adults, which can be sent singly or in groups in plastic vials equipped with water-drenched gauze and some sugar. To collect pupae, eggs are placed in sufficient dung directly onto dry sand in a larger plastic container. The larvae will hatch, feed on the dung, and eventually crawl down into the sand to pupate. After 8–12 d, they can be removed by sieving. Pupae can be mailed in a sturdy shallow Petri dish with a bit of sand. Still, they should not be exposed to temperatures exceeding 25° C.

**Figure 3:**
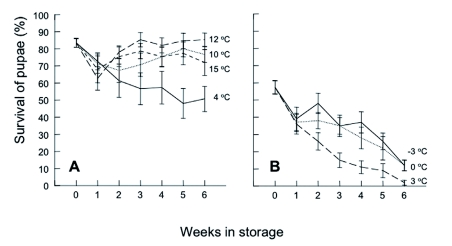
Percent survival (± SD) of *S. stercoraria* pupae after storage for periods of 1 to 6 weeks at different temperatures. (A) Experiment 1: storage at 4 to 15° C; (B) Experiment 2; storage at 3 to -3° C. High quality figures are available online.

Blanckenhorn (1998b) showed that larvae from Swiss populations facing low temperature (10° C) and photoperiod combinations (indicating the advent of winter) will enter winter diapause as pupae for up to 5 months, after which adults will emerge at survival rates comparable to direct development. In general, there may be occasions when it is desirable to store at cool temperatures pupae generated from adults held at room temperature, until needed for colony expansion or experimental studies. To test the effect of cold storage on survival (novel data presented in the Results section), 15 replicates of fly pupae (*n* = 10 pupae per replicate), aged 24–48 h, were stored at 4, 10, 12, or 15° C for periods of 1, 2, 3, 4, 5, or 6 weeks. Upon completion of the storage period, pupae were removed and held at 20° C for an additional week. Numbers of emergent flies were recorded. Puparia not producing flies were dissected to determine whether pupae were dead or in a state of arrested development. As a control, 20 replicates of pupae were held continuously at 20° C. Pupae used for different combinations of storage time and temperature, and for the control replicates, were from the same generational cohort. Flies occasionally emerged while in storage at temperatures of 10, 12 and 15° C. This is not surprising, as individuals held at temperatures exceeding 12° C are unlikely to enter pupal diapause (Blanckenhorn 1998b), so adults are expected to emerge from pupae after roughly half the development time listed in Table 1 for that particular temperature. Hence, the experiment was repeated using storage temperatures of-3, 0 and 3° C, with 15 (rather than 20) replicates of pupae held at 20° C for use as controls. No flies emerged during storage at these lower temperatures. The results presented below indicate that larvae developing at warm temperatures will not be properly prepared physiologically for pupal diapause, and thus keeping the resulting pupae at cold temperatures for extended periods of time will inevitably result in significant mortality (Figure 3). Longer storage of non-diapausing pupae in the fridge is therefore not feasible.

### Processing cow dung for experimentation

Cow dung can be collected in large quantities from the pasture in the field, mixed thoroughly in larger vats or buckets in the laboratory (to reduce variation in dung quality), and frozen in portions of suitable size for extended periods of time to be used later, marking the date on the pot lid or zip-lock bag with a waterproof pen. Any parasites or other organisms in the dung can be killed by freezing the dung first at -20° C and thereafter at -80° C for a minimum of 1 week. (Freezing at merely -20° C might not kill some parasites, and storing fresh dung at -80° C without an intermediate -20° C step causes most freezers to trip their alarms because the samples do not cool quickly enough.) A ca. 5 cm gap should be left at the top of the dung container to allow for expansion as the dung freezes, or the container may crack. When needed, dung can be thawed overnight and, if necessary, mixed with some warm water to obtain a looser consistency. If needed more quickly, dung containers can be submerged in hot water or defrosted in a microwave oven.

Dung quality (i.e. water, nutrient, parasite or medication content, etc.) has great impact on larval development and ultimately the fitness of the individual fly. Unfortunately, dung quality usually cannot be controlled effectively over longer periods of time. For example, cows fed on dried hay over the winter produce dung of a lower food quality than those fed on summer grass. Therefore, the same, homogenized and thoroughly mixed dung batch should be used for any particular experiment. Because insecticidal residues from veterinary drugs may be excreted in dung of treated livestock for at least 3 months after application (e.g. Floate 1998; Floate et al. 2008), dung is best collected from animals that have not been treated with parasiticides in the previous six months.

When testing the effect of pharmaceuticals on *S. stercoraria* according to the OECD guideline (2008), the dung should have been frozen for at least 1 week (preferably longer). Dung samples should be taken to determine its moisture and pH. The test substance in question has to be mixed thoroughly into the dung, usually at various, roughly logarithmicly-scaled concentrations. If chemicals volatile solvent (typically acetone or ethanol) and mixed thoroughly for ca. 10 minutes. The two mandatory control treatments are inoculated with a known amount of solvent (solvent control) and with an equivalent amount of water only (untreated control). When a solvent carrier is used, it must be allowed to fully evaporate for at least 4 hours at room temperature before the test organisms are added. All test concentrations must be given on a dry weight basis in order to ensure comparability of the results from different studies.

## Results

As a guide, means and ranges for all relevant, standard life history traits stemming from previously published sources are reported here, referring to primarily one Swiss population (Fehraltorf near Zurich). These data, stemming from lab and field experiments, are summarized in Table 1, which includes the original references.

### Cold storage of pupae

For the experiment in which pupae were stored at temperatures of 4, 10, 12 and 15° C, survival (= fly emergence) of control pupae was 83.5% ± 2.5 (SD; Figure 3A). Using linear regression, no effect of weeks in storage was detected for the survival of pupae held at temperatures of 10° C (*F*_1, 108_ = 0.01; p = 0.911) or 15° C (*F*_1, 108_ = 0.53; p = 0.466). A weak, though overall positive, effect of storage (*F*_1, 108_ = 3.99; p = 0.048) was detected for pupae held at 12° C. This can be attributed to the unexpectedly low survival of pupae removed from storage after one week; survival of pupae held for 6 weeks at 12°C (85.3% ± 3.8) did not differ from that of control pupae. However, at 4° C (*F*_1, 108_ = 17.76; p < 0.001) survival declined to 50.7% ± 7.1 after 6 weeks in storage. Dissection of puparia from which flies did not emerge revealed pupae to be dead and desiccated rather than in a state of arrested development.

For the experiment in which pupae were stored at temperatures of -3, 0 and 3° C, survival of control pupae was 57.3% ± 37.1 (Figure 3B). At all three temperatures survival declined with time to 12.0% ± 2.9 after storage for 6 weeks at -3 and 0° C, and to 2.0% ± 1.3 at 3° C (*F*_1, 73_ = 46.0 to 128.6; p < 0.001).

## Conclusions

Baseline information and summary data are provided here on the natural history, field and laboratory handling of *S. stercoraria* for the benefit of the international scientific community, as well as for government regulators or testing agencies that use this fly for eco-toxicological testing. Based on the availability of data and experience with this fly, the information primarily reflects the situation of Swiss dung fly populations. It is clear that variation in the life history of this fly will be considerable given its wide distribution on at least three continents (North America, Asia, Europe) in climates ranging from sub-arctic to Mediterranean. Systematic comparisons of geographic, latitudinal and altitudinal populations on a worldwide scale are consequently necessary and underway.

## References

[bibr01] Amano K (1983). Studies on the intraspecific competition in dung breeding flies. I. Effects of larval density on the yellow dung fly.. *Japanese Journal of Sanitary Zoology*.

[bibr02] Arthur BI, Sbilordo SH, Pemberton AJ, Ward PI (2008). The anatomy of fertilization in the yellow dung fly *Scathophaga stercoraria*.. *Journal of Morphology*.

[bibr03] Bernasconi MV, Pawlowski J, Valsangiacomo C, Piffaretti JC, Ward PI (2001). Phylogeny of the genus *Scathophaga* (Diptera: Scathophagidae) inferred from mitochondrial DNA sequences.. *Canadian Journal of Zoology*.

[bibr04] Blanckenhorn WU (1997). Altitudinal life history variation in the dung flies *Scathophaga stercoraria* and *Sepsis cynipsea*.. *Oecologia*.

[bibr05] Blanckenhorn WU (1998a). Adaptive phenotypic plasticity in growth rate and diapause in the yellow dung fly.. *Evolution*.

[bibr06] Blanckenhorn WU (1998b). Altitudinal differentiation in diapause response in two species of dung flies.. *Ecological Entomology*.

[bibr07] Blanckenhorn WU (1999). Different growth responses to food shortage and temperature in three insect species with similar life histories.. *Evolutionary Ecology*.

[bibr08] Blanckenhorn WU (2000). Temperature effects on egg size and their fitness consequences in the yellow dung fly.. *Evolutionary Ecology*.

[bibr09] Blanckenhorn WU (2002). The consistency of heritability estimates in field and laboratory in the yellow dung fly.. *Genetica*.

[bibr10] Blanckenhorn WU, Ananthakrishnan TN, Whitman DW (2009). Causes and Consequences of phenotypic plasticity in body size: The case of the yellow dung fly *Scathophaga stercoraria* (Diptera: Scathophagidae).. *Phenotypic Plasticity of Insects: Mechanisms and Consequences*..

[bibr11] Blanckenhorn WU, Henseler C (2005). Temperature-dependent ovariole and testis maturation in the yellow dung fly.. *Entomologia Experimentalis et Applicata*.

[bibr12] Blanckenhorn WU, Henseler C, Burkhard DU, Briegel H (2001). Summer decline in populations of the yellow dung fly: Diapause or quiescence?. *Physiological Entomology*.

[bibr13] Blanckenhorn WU, Llaurens V (2005). Effects of temperature on cell size and number in the yellow dung fly *Scathophaga stercoraria*.. *Journal of Thermal Biology*.

[bibr14] Blanckenhorn WU, Viele STN (1999). Foraging in yellow dung flies: Testing for a small male time budget advantage.. *Ecological Entomology*.

[bibr15] Cotterell GS (1920). The life history and habits of the yellow dung fly (*Scathophaga stercoraria*): A possible blow-fly check.. *Proceedings of the Zoological Society London*.

[bibr16] Floate KD (1998). Off-target effects of ivermectin on insects and on dung degradation in southern Alberta, Canada.. *Bulletin of Entomological Research*.

[bibr17] Floate KD (2007). Endectocide residues affect insect attraction to dung from treated cattle: Implications for toxicity tests.. *Medical and Veterinary Entomology*.

[bibr18] Floate KD, Bouchard P, Holroyd GL, Poulin RG, Wellicome TI (2008). Does doramectin use on cattle indirectly affect the endangered Burrowing Owl?. *Rangeland Ecology and Management*.

[bibr19] Floate KD, Coghlin PC (2010). No support for fluctuating asymmetry as a biomarker of chemical residues in livestock dung.. Canadian Entomologist (*in press*)..

[bibr20] Foster W (1967). Hormone-mediated nutritional control of sexual behavior in male dung flies.. *Science*.

[bibr21] Gibbons DS (1980a). Prey consumption, mating, and egg production in *Scathophaga* species (Dipt., Scathophagidae) in the laboratory.. *Entomologist's Monthly Magazine*.

[bibr22] Gibbons DS (1980b). The feeding habits of *Scathophaga stercoraria* (Diptera: Scathophagidae) on cow pats.. *Entomologist's Monthly Magazine*.

[bibr23] Gibbons DS (1987). The causes of seasonal changes in numbers of the yellow dung fly *Scathophaga stercoraria*.. *Ecological Entomology*.

[bibr24] Gorodkov KB, Soós A, Papp L (1986). Scathophagidae.. *Catalogue of Palaearctic Diptera*..

[bibr25] Hammer O (1941). Biological and ecological investigations on flies associated with pasturing cattle and their excrement.. *Videnskabelige Meddelelser fra Dansk Naturhistorisk Forening*.

[bibr26] Hirschberger P, Degro HN (1996). Oviposition of the dung beetle *Aphodius ater* in relation to abundance of yellow dung fly larvae (*Scathophaga stercoraria*).. *Ecological Entomology*.

[bibr27] Holter P (1979). Effect of dung-beetles (*Aphodius* spp) and earthworms on the disappearance of cattle dung.. *Oikos*.

[bibr28] Hosken DJ, Blanckenhorn WU, Ward PI (2000). Developmental stability in yellow dung flies *(Scathophaga stercoraria*): Fluctuating asymmetry, heterozygosity and environmental stress.. *Journal of Evolutionary Biology*.

[bibr29] Hosken DJ, Garner TWJ, Ward PI (2001). Sexual conflict selects for male and female reproductive characters.. *Current Biology*.

[bibr30] Hosken DJ, Ward PI (1999). Female accessory reproductive gland activity in the yellow dung fly, *Scathophaga stercoraria* (Diptera: Scathophagidae).. *Journal of Insect Physiology*.

[bibr31] Jann P, Blanckenhorn WU, Ward PI (2000). Temporal and microspatial variation in the intensities of natural and sexual selection in the yellow dung fly *Scathophaga stercoraria*.. *Journal of Evolutionary Biology*.

[bibr32] Jann P, Ward PI (1999). Maternal effects and their consequences for offspring fitness in the yellow dung fly.. *Functional Ecology*.

[bibr33] Kraushaar U, Blanckenhorn WU (2002). Population variation in sexual selection and its effect on body size allometry in two species of flies with contrasting sexual size dimorphism.. *Evolution*.

[bibr34] Kraushaar U, Goudet J, Blanckenhorn WU (2002). Geographical and altitudinal population genetic structure of two dung fly species with contrasting mobility and temperature preference.. *Heredity*.

[bibr35] OECD (Organisation for Economic Cooperation and Development). (2008). *Guideline for the Testing of Chemicals. Draft: Determination of Developmental Toxicity of a Test Chemical to Dipteran Dung Flies (Scathophaga stercoraria L. (Scathophagidae) and Musca autumnalis De Geer (Muscidae))*.. http://www.oecd.org/dataoecd/52/58/38207891.pdf.

[bibr36] Otronen M (1996). Effects of seasonal variation in the operational sex ratio and population density on the mating success of different sized and aged males in the yellow dung fly *Scathophaga stercoraria*.. *Ethology Ecology and Evolution*.

[bibr37] Parker GA (1970a). The reproductive behaviour and the nature of sexual selection in *Scatophaga stercoraria* L. (Diptera: Scatophagidae) I. Diurnal and seasonal changes in population density around the site of mating and oviposition.. *Journal of Animal Ecology*.

[bibr38] Parker GA (1970b). The reproductive behaviour and the nature of sexual selection in *Scatophaga stercoraria* L. (Diptera: Scatophagidae). II. The fertilization rate and the spatial and temporal relationships of each sex around the site of mating and oviposition.. *Journal of Animal Ecology*.

[bibr39] Parker GA (1970c). Sperm competition and its evolutionary effect on copula duration in the fly *Scatophaga stercoraria*.. *Journal of Insect Physiology*.

[bibr40] Parker GA, Krebs JR, Davies NB (1978). Searching for mates.. *Behavioural Ecology*.

[bibr41] Reim C, Teuschl Y, Blanckenhorn WU (2006). Size-dependent effects of larval and adult food availability on reproductive energy allocation in the yellow dung fly.. *Functional Ecology*.

[bibr42] Römbke J, Barrett K, Gray J, Blanckenhorn WU, Jochmann R, Knäbe S, Lehmhus J, Rosenkranz B, Sekine T, Scheffczyk A, Schmidt T, Sharpies A, Davies N (2009). Lethal and sublethaltoxic effects of a test chemical (ivemectin) on the Yellow Dung Fly *Scathophaga stercoraria* based on a standardsized international ring test.. *Environmental Toxicology & Chemistry*.

[bibr43] Sasaki H (1984). A comparative study of the ecology of yellow dung flies (Diptera: Scathophagidae). I. Predatory ability and predatory behaviour.. *Japanese Journal of Sanitary Zoology*.

[bibr44] Sigurjónsdóttir H, Snorrason SS (1995). Distribution of male yellow dung flies around oviposition sites: The effect of body size.. *Ecological Entomology*.

[bibr45] Simmons LW (2001). *Sperm Competition and its Evolutionary Consequences in the Insects*..

[bibr46] Sommer C, Steffansen B, Overgaard Nielsen B, Gronvold J, Vagn-Jensen KM, Brochner J, Jespersen J, Springborg J, Nansen P (1992). Ivermectin excreted in cattle dung after subcutaneous injection or pour-on treatment: Concentrations and impact on dung fauna.. *Bulletin of Entomological Research*.

[bibr47] Sowig P, Himmelsbach R, Himmelsbach W (1997). Predator-prey relationship between insect larvae: Growth of *Sphaeridium* larvae (Coleoptera: Hydrophilidae) under time constraints through predation on *Musca autumnalis* maggots (Diptera: Muscidae).. *Canadian Journal of Zoology*.

[bibr48] Stone A, Sabrosky CW, Wirth WW, Foote RH, Coulson (1965). *A Catalog of the Diptera of America North of Mexico*..

[bibr49] Strong L, James S (1992). Some effects of rearing the yellow dung fly *Scathophaga stercoraria* in cattle dung containing Ivermectin.. *Entomologia Experimentalis et Applicata*.

[bibr50] Swaddle JP (1997). Developmental stability and predation success in an insect predatorprey system.. *Behavioral Ecology*.

[bibr51] Teuschl Y, Reim C, Blanckenhorn WU (2007). Correlated responses to artificial body size selection in growth, development, phenotypic plasticity and juvenile viability in yellow dung flies.. *Journal of Evolutionary Biology*.

[bibr52] Ward PI (2000). Cryptic female choice in the yellow dung fly *Scathophaga stercoraria*.. *Evolution*.

[bibr53] Ward PI, Simmons LW (1990). Short-term changes in the numbers of the yellow dung fly, *Scathophaga stercoraria*.. *Ecological Entomology*.

[bibr54] Webb L, Beaumont DJ, Nager RG, McCracken DI (2007). Effects of avermectin residues in cattle dung on yellow dung fly *Scathophaga stercoraria* (Diptera: Scathophagidae) populations in grazed pastures.. *Bulletin of Entomological Research*.

[bibr55] Werner D, Mann DJ, Pont AC (2006). Notes on predation by Scathophagidae (Diptera) on Simulidae (Diptera).. *Entomologist's Monthly Magazine*.

